# Testicular toll with viral intrusion: Molecular insights into the impact of chronic HIV infection on male fertility

**DOI:** 10.1002/ctm2.70815

**Published:** 2026-09-11

**Authors:** Tousif Ahmed Hediyal, Elizabeth M. Stone, Omar Shukri, Victoria L. Schaal, Amin Foroughi‐Nezhad, Murali Devanaboyina, Josef S. Crenshaw, Amir Elrefaie, Sree Kolli, Camryn N. Davis, Dinesh Pradhan, Sowmya V. Yelamanchili, Gurudutt Pendyala

**Affiliations:** ^1^ Department of Anesthesiology University of Nebraska Medical Center (UNMC) Omaha Nebraska USA; ^2^ Department of Pathology, Microbiology & Immunology University of Nebraska Medical Center (UNMC) Omaha Nebraska USA; ^3^ Department of Genetics, Cellular Biology, and Anatomy UNMC Omaha Nebraska USA; ^4^ National Strategic Research Institute, UNMC Omaha Nebraska USA; ^5^ Child's Health Research Institute Omaha Nebraska USA

**Keywords:** CASA, CatSper channels, HIV, male infertility, sperm morphology

## Abstract

**Background:**

Human immunodeficiency virus (HIV) infection remains a major global health concern, compromising immune function and increasing susceptibility to opportunistic infections. Over the past two decades, fertility desire among people living with HIV has remained low due to poor health, fear of transmission and restrictive reproductive policies. In men, particularly those in advanced stages of infection, HIV has been associated with profound alterations in semen quality, including reduced ejaculate volume, impaired sperm motility, abnormal morphology and increased incidence of sperm aneuploidy factors that collectively contribute to male infertility.

**Methods:**

This study explored the molecular mechanisms of HIV‐associated male infertility using a preclinical HIV‐1 transgenic (Tg) rat model. Real‐time PCR (RT‐PCR) and protein expression analyses were performed to evaluate the expression of cation channels of sperm (CatSper 1‐4), along with mitochondrial and inflammatory gene networks. Histopathological examination, computer‐assisted semen analysis (CASA), nuclear morphology assessment, and transcriptomic sequencing were used to characterize structural, functional, and molecular alterations in the testes and sperm of Tg animals.

**Results:**

RT‐PCR and protein expression analyses revealed marked downregulation of CatSper1‐4 channels, along with dysregulation of mitochondrial and inflammatory gene networks in the testes of Tg animals. Histopathological examination further demonstrated structural disruptions within the seminiferous tubules of Tg rats. Functional assessments using CASA and nuclear morphology analysis confirmed reduced motility and abnormal sperm morphology in Tg rats. Complementary transcriptomic sequencing identified novel gene signatures and disrupted pathways associated with testicular dysfunction in Tg rats.

**Conclusion:**

Collectively, our findings demonstrate that HIV‐1 transgene expression impairs sperm production and function at both mRNA and protein levels by disrupting ion channel regulation and perturbing broader molecular pathways, ultimately contributing to male infertility. This study provides clinically relevant insights into reproductive complications in HIV‐infected individuals and may inform future therapeutic strategies.

**Key points:**

- HIV‐1 transgene expression impairs testicular architecture and spermatogenesis.
- CatSper 1‐4 channels downregulation is associated with impaired sperm function.
- Mitochondrial dysfunction and altered immune signaling pathways accompany abnormal sperm functions.
- HIV‐1 transgenes induce profound abnormalities in sperm morphology, motility, and widespread transcriptional remodeling of reproductive pathways.

## Graphical abstract

The molecular mechanisms contributing to impaired fertility in HIV+ males remain poorly defined. Using a human immunodeficiency virus (HIV) transgenic (Tg) rat model, we demonstrate key structural and functional sperm regulators including cation channel of sperm (CatSper)1–4 channels and mitochondrial biogenesis genes were markedly downregulated, accompanied by corresponding abnormalities in testicular architecture and sperm motility.

Proposed model illustrating how HIV‐1 transgene disrupts male reproductive function. HIV‐1 transgene induces severe testicular dysfunction marked by impaired spermatogenesis, seminiferous tubule degeneration, abnormal sperm morphology and reduced motility. These abnormalities are associated with downregulation of CatSper calcium‐channel subunits and mitochondrial dysfunction, disrupting Ca^2^^+^ signaling and ATP production. Consequently, sperm hyperactivation, capacitation and fertilization capacity are compromised, reducing male fertility. *Figure generated using Figure Labs AI software under licensure*.

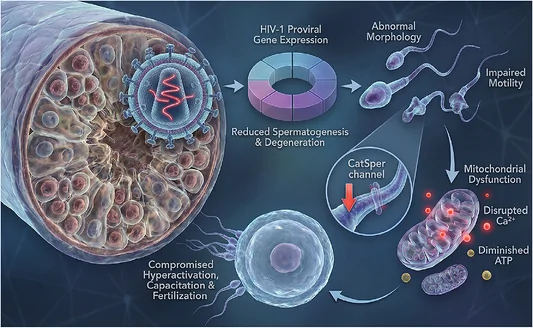

## 1 INTRODUCTION

Human immunodeficiency virus (HIV) is a retrovirus that replicates through reverse transcription and that causes acquired immunodeficiency syndrome (AIDS).^1^ It remains a primary global health concern for both the individuals and healthcare system.^2^ According to the 2023 United Nations Program on HIV/AIDS (UNAIDS) report, approximately 39.9 million people globally were living with HIV, with about 1.5 million people newly infected annually. Of these, 38.6 million were individuals of reproductive age (15+ years), including 20 million women and 18.1 million men. Widespread access to life‐saving antiretroviral therapy (ART) has dramatically reduced AIDS‐related mortality to its lowest since its peak in 2004.^3^, ^4^ Despite these advances, fertility intention among people living with HIV has remained low over the past two decades, influenced by concerns about personal health, risk of partner or vertical transmission and restrictive reproductive polices in many regions.^5^

Following infection, HIV rapidly enters the testes by crossing the blood‐testes barrier (BTB), establishing a reservoir for latent infection.^6^ Although HIV typically does not directly damage germ cells or alter testicular morphology, it targets testicular lymphocytes.^7^ The testes can preserve productive infection by HIV‐1, with CD68+ macrophages serving as the virus‐replicating cells.^8^ Because the testes act as a sanctuary site that is relatively resistant to ART and because semen remains the primary vector for HIV‐1 transmission, understanding the virus's effects on the male reproductive tract is essential.

Clinically, HIV‐infected men exhibit higher rates of orchitis, oligospermia, hypogonadism, impotence and diminished libido. Up to 60% of men with advanced HIV disease report impaired erectile function and ejaculation.^9^ Studies have revealed reduced ejaculate volume, sperm motility and sperm count, as well as increased abnormal sperm morphology, altered seminal fluid pH (6.5–6.9) and increased viscosity in asymptomatic HIV‐positive men.^10^ Decreases in total ejaculate volume and sperm concentration have been likewise reported.^11^ While reductions in sperm motility may be partly attributed to ART, increased viscosity and decreased volume are associated with functional impairment of the seminal vesicles and prostate. Because the cation channel of sperm (CatSper) calcium channel complex, particularly CatSper subunit α 1–4, is essential for sperm motility; HIV‐1‐induced alterations to this pathway may contribute to impaired sperm function. Emerging evidence suggests that HIV‐1 may disrupt CatSper regulation, sperm morphology and testicular architecture, although the precise mechanisms remain unclear.

Mitochondrial dysfunction represents another key contributor to HIV‐associated impairments in sperm quality. In addition to supplying ATP required for sperm motility, mitochondria play a critical role in regulating intracellular calcium homeostasis,^12^ which is pivotal for proper CatSper channel activity. HIV‐induced mitochondrial injury and apoptotic pathways^13^ may therefore lead to dysregulated calcium signaling and impaired CatSper function, exacerbating defects in sperm motility and morphology.

Despite growing evidence linking HIV infection to impairments in testicular function, spermatogenesis, ion‐channel activity and mitochondrial homeostasis, the mechanisms integrating these pathological processes remain incompletely understood. In the present study, we utilized a well‐established HIV‐1 Tg rat model that recapitulates the chronic effects of persistent HIV‐1 gene expression to investigate the molecular, structural and functional pathways underlying male reproductive dysfunction. By examining the interplay among CatSper channel regulation, mitochondrial bioenergetics, inflammation and spermatogenesis, this work provides new insight into the mechanisms through which chronic HIV‐1 protein exposure disrupts male reproductive health and identifies potential molecular pathways for therapeutic intervention and fertility preservation.

## 2 RESULTS

### 2.1 HIV‐1 transgene expression reduces testicular size, hormone levels and sperm count in HIV‐1 Tg rats

**FIGURE 1:**
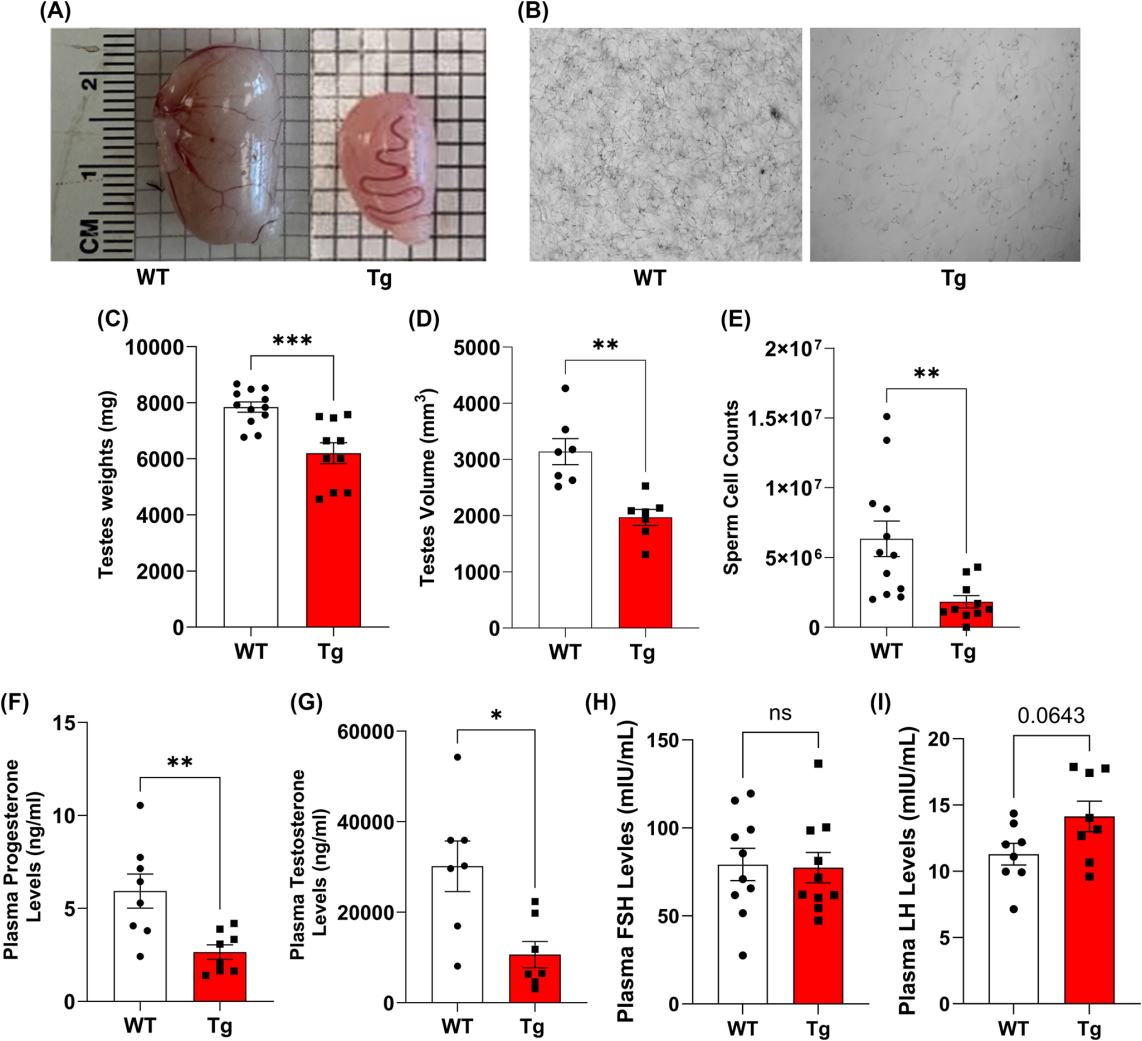
Effects of human immunodeficiency virus (HIV)‐1 transgene on male reproductive parameters. Gross morphological analysis shows significant reduction in testes weight and testes volume of transgenic (Tg) rats compared with wild type (WT) controls (A, C, D). Sperm quantification likewise revealed a significant decrease in total sperm count in Tg animals relative to WT (B, E). Hormonal profiling demonstrated substantially lower plasma progesterone and testosterone levels in Tg rats. Follicle‐stimulating hormone (FSH) levels are unchanged between both groups, however, luteinising hormone (LH) was trending upwards for the Tg animals (F–I). Data are presented as mean ± SEM, and statistical significance was determined using an unpaired *t*‐test with Welch's correction. *p < .05, **p < .01, ***p < .001. *n* = 10–12 samples were used for the analysis.

Testicular development and function are critical determinants of male reproductive health.^14^ Common indicators of testicular integrity include organ weight and volume, circulating hormone levels and sperm production.^15^, ^16^ In this study, gross morphological analysis of testicular tissue (Figure 1A) revealed that Tg rats exhibited a significant (*p* < .001) reduction in testicular weight and testicular volume (*p* < .01) compared with wild type (WT) controls (Figure 1C,D).

Consistent with these morphological deficits, sperm quantification showed a significant (*p* < .01) decrease in sperm count in Tg rats relative to WT animals (Figure 1B,E). Hormonal profiling further demonstrated marked reductions in progesterone (*p* < .01) and testosterone (*p* < .05) levels in both testicular plasma and tissue lysates of Tg animals compared with WT rats (Figure 1F,G). Reduced progesterone levels, a precursor in testosterone biosynthesis, are indicative of impaired steroidogenic capacity and compromised reproductive function. To further investigate whether these hormonal alterations were associated with disruptions of the hypothalamic–pituitary–gonadal axis, plasma follicle‐stimulating hormone (FSH) and luteinising hormone (LH) levels were measured. No significant difference in FSH levels was observed between WT and Tg animals (Figure 1H). However, LH levels trended towards an increase in Tg rats relative to the WT animals (Figure 1I). As FSH and LH are the key regulators of testicular steroidogenesis and spermatogenesis, respectively, these findings indicate that reductions in the testosterone and progesterone levels occurred in the absence of marked alterations in circulating gonadotropin levels. Taken together, these findings demonstrate that HIV‐1 transgene expression is associated with reduced testicular size, impaired sperm production and decreased gonadal steroid hormone levels, while circulating gonadotropin levels remain largely preserved.

### 2.2 Histopathology reveals seminiferous tubule degeneration in Tg rats

**FIGURE 2:**
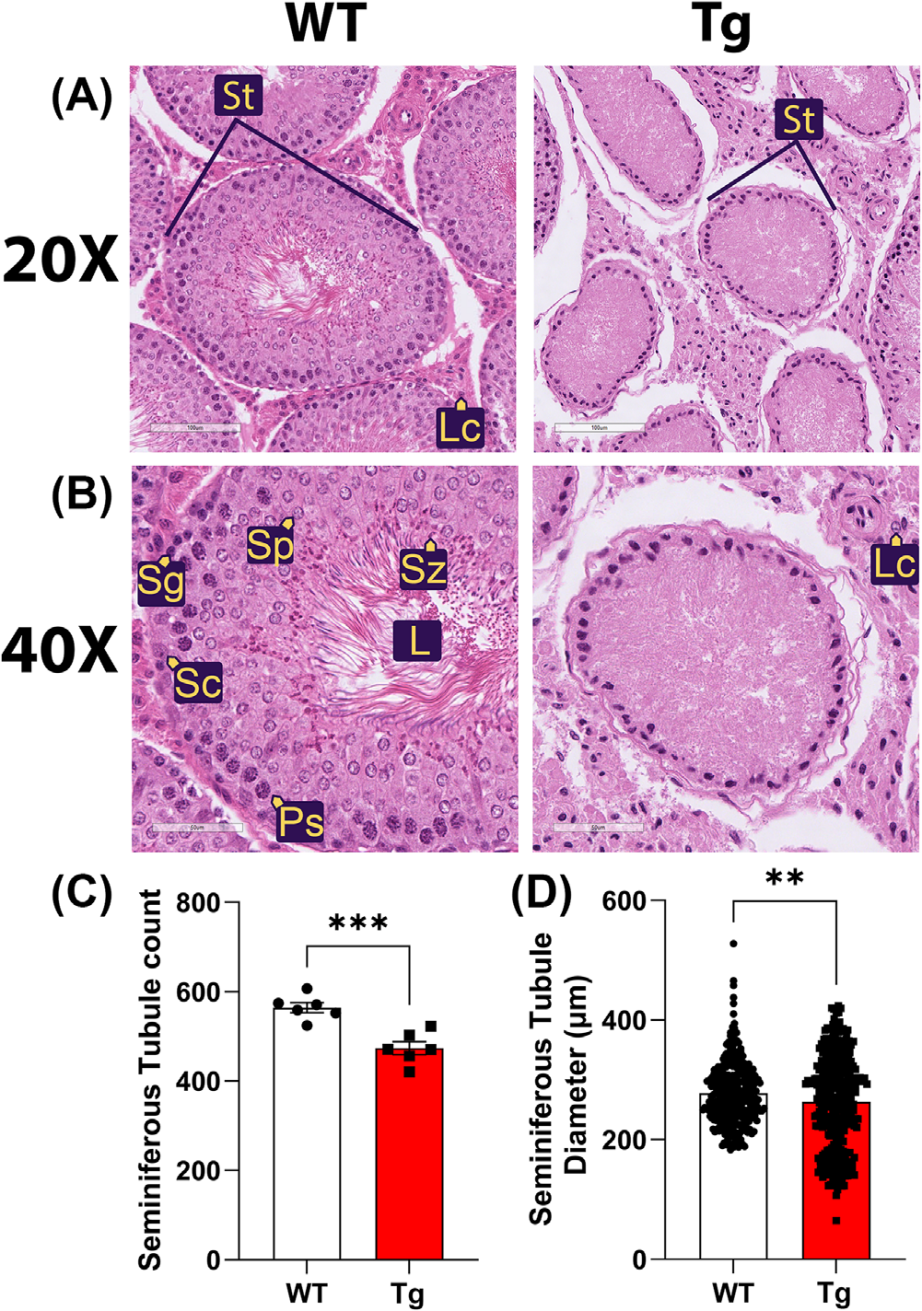
Effect of human immunodeficiency virus (HIV)‐1 transgene on testicular architecture. Representative haematoxylin and eosin (H&E)‐stained sections of testes at 20× and 40× magnification (A, B). Wild type (WT) testes exhibit normal seminiferous tubule (St) organisation with well‐defined germ cell layers, including spermatogonia (Sg), primary spermatocytes (Ps), spermatids (Sp) and spermatozoa (Sz), along with Sertoli cells (Sc) and an open lumen (L). Leydig cells (Lc) are clearly visible within the interstitial spaces. In contrast, transgenic (Tg) testes display disrupted seminiferous tubule architecture characterised by reduced germ cell layers, diminished cellular organisation and smaller luminal spaces. Quantitative analysis shows a significant reduction in the number of seminiferous tubules per section in Tg rats compared with WT controls (C). Similarly, seminiferous tubule diameter is markedly decreased in Tg animals (D). Data are presented as mean ± SEM, and statistical significance was evaluated using an unpaired *t*‐test with Welch's correction. *p < .05, **p < .01. *n* = 6 samples were used for analysis.

Histological evaluation of testicular sections revealed marked structural differences between WT and Tg rats. At both 20× and 40× magnification, WT testes displayed well‐organised seminiferous tubules containing complete spermatogenic cell layers, including spermatogonia, spermatocytes, spermatids and luminal spermatozoa. In contrast, Tg testes exhibited noticeably smaller seminiferous tubules with reduced germ cell populations and diminished luminal sperm. Leydig cells were also less abundant and appeared more dispersed in Tg animals tissue compared to WT (Figure 2A,B). Quantitative morphometric analysis demonstrated a significant (*p *< .001) reduction in seminiferous tubules in Tg testes relative to WT (Figure 2C). Also, seminiferous tubule diameter was significantly (*p* < .01) decreased in Tg animals, consistent with disrupted spermatogenic architecture (Figure 2D). Collectively, these findings indicate that expression of the HIV‑1 transgene is associated with substantial testicular structural degeneration, impaired seminiferous tubule integrity and compromised spermatogenesis.

### 2.3 The CatSper channel complex is disrupted in HIV‐1 Tg rats

**FIGURE 3:**
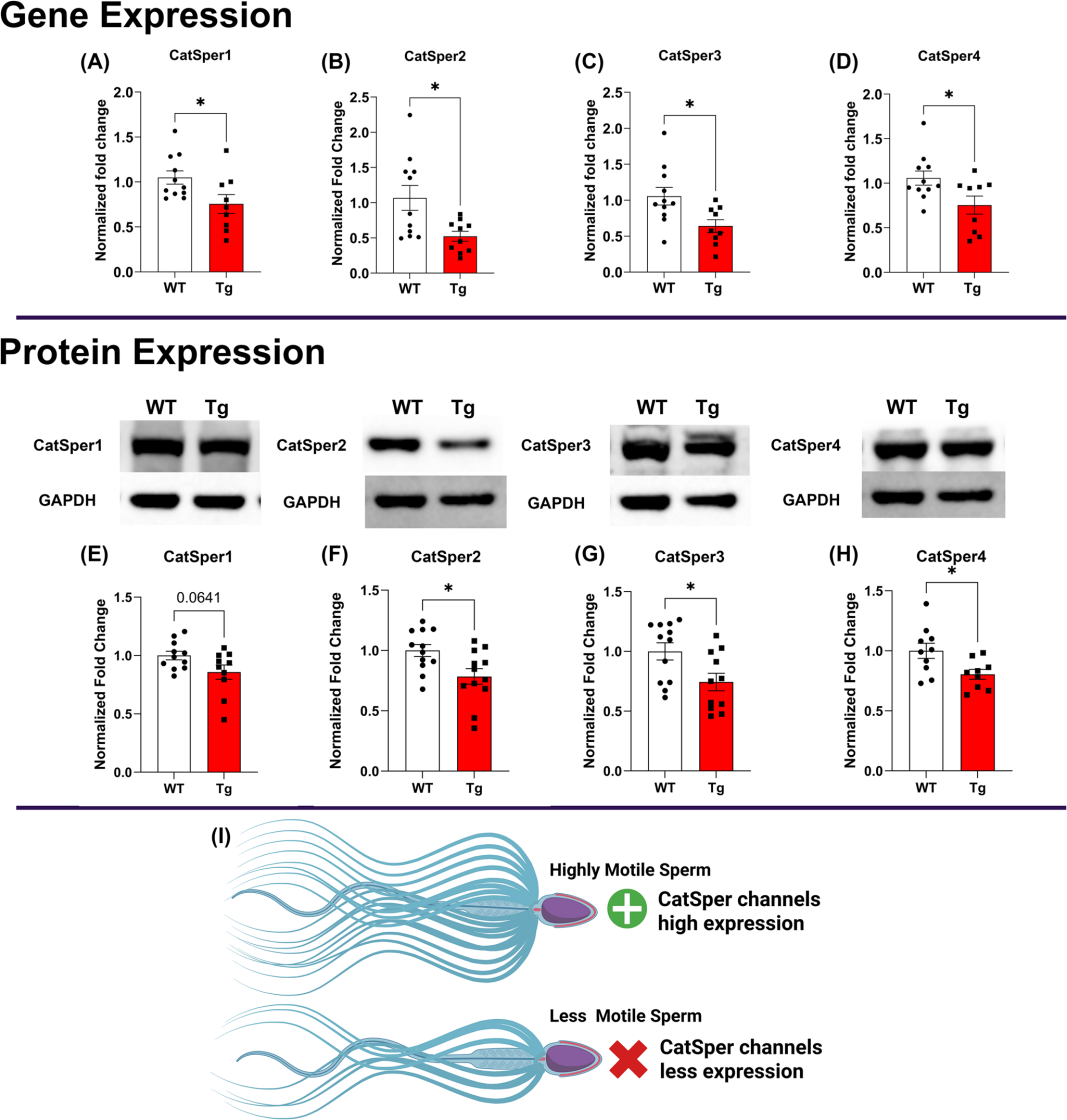
Effect of human immunodeficiency virus (HIV)‐1 transgene on cation channels of sperm (CatSper). Normalized fold‐change expression levels of CatSper1–4 are significantly reduced in transgenic (Tg) animals compared with wild type (WT) controls (A–D). These reductions were further validated through Western blot, also showing reduced protein expression of CatSper1–4 (E–H). Panel (I) provides a schematic representation illustrating the functional implications of altered CatSper expression: high CatSper levels support robust sperm motility, whereas reduced CatSper expression is associated with diminished motility and impaired sperm function. Data are presented as mean ± SEM, and statistical significance was determined using an unpaired *t*‐test with Welch's correction. *p < .05, **p < .01. *n* = 10–12 samples were used for the analysis.

The CatSper complex is essential for regulating calcium influx required for sperm hyperactivation, chemotaxis and the acrosome reaction processes critical for successful fertilization.^17^ Real‐time quantitative PCR (RT‐qPCR) analysis revealed that Tg rats exhibit a significant (*p* < .05) reduction in CatSper1–4 expression compared with WT animals (Figure 3A–D). Consistent with the gene expression data, protein levels of CatSper2–4 were significantly reduced (*p* < .05) in HIV‐1 Tg rats compared with WT controls. Although CatSper1 protein expression did not reach statistical significance, it exhibited a strong trend towards downregulation (*p* = .0641) in Tg animals (Figure 3E–H). The concordant reductions observed at both the transcript and protein levels support impaired expression of the CatSper channel complex, suggesting disruption of the calcium signaling mechanisms essential for normal sperm motility and function. Given the central role of CatSper in driving sperm hyperactivation, guiding chemotactic movement and facilitating acrosomal penetration, the reduced expression of these channels in Tg animals may contribute to the impaired sperm parameters and reproductive abnormalities identified in this study (Figure 3I). The coordinated downregulation of multiple CatSper subunits supports the notion that HIV‐1 transgene expression disrupts calcium signaling pathways essential for normal sperm function and may represent an important mechanism underlying male reproductive dysfunction.

### 2.4 HIV‐1 transgene expression disrupts sperm nuclear morphology and motility

**FIGURE 4:**
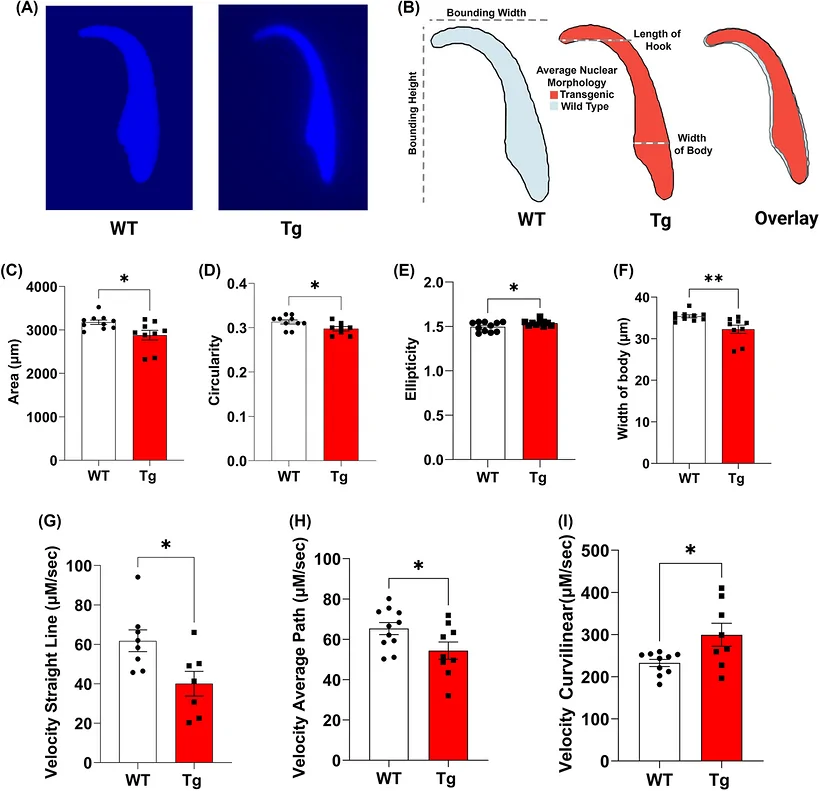
Effect of human immunodeficiency virus (HIV)‐1 transgene on sperm morphology and motility. DAPI‐stained sperm from transgenic (Tg) rats exhibit marked alterations in sperm head architecture compared with wild type (WT) controls (A, B). Quantitative analysis of more than 500 sperm nuclei per group revealed significant differences in nuclear morphology, including area (C), circularity (D), ellipticity (E) and body width (F). Computer‐assisted semen analysis (CASA)‐based motility assessment demonstrated that Tg rats display significantly reduced straight line velocity (VSL) (G) and average path velocity (VAP) (H) yet increased curvilinear velocity (VCL) (I) relative to WT animals. Data are presented as mean ± SEM, and statistical significance was determined using an unpaired *t*‐test with Welch's correction. *p < .05, **p < .01. *n* = 8–12 samples were used for the analysis.

Structural features of the sperm head, particularly nuclear shape and organization, serve as important indicators of underlying reproductive pathologies. Increased abnormalities in sperm head morphology are often associated with DNA fragmentation and compromised structural integrity of the sperm cell. To assess whether HIV‐1 transgene affects sperm architecture, geometric nuclear morphology analysis was performed on 4’,6‐Diamidino‐2‐phenylindole (DAPI)‐stained spermatozoa. Qualitative assessment revealed that sperm from Tg rats exhibited visibly altered head shapes compared with WT controls (Figure 4A,B). Quantitative analysis of more than 500 sperm nuclei per group demonstrated significant alterations in several morphological parameters in Tg animals, including nuclear area (*p* < .05), circularity (*p* < .01), ellipticity (*p* < .05) and body width (*p* < .05; Figure 4C–F). These findings indicate that HIV‐1 transgene expression induces pronounced abnormalities in sperm head morphology, potentially compromising fertilization capacity in Tg rats.

Sperm motility was assessed using CASA on freshly collected, swim‐up sperm incubated in M2 medium, following the recommended guidelines for mammalian sperm analysis. Tg rats displayed significant reductions in key motility parameters including straight line velocity (VSL; *p* < .05) and average path velocity (VAP; *p* < .05), plus an increase in curvilinear velocity (VCL; *p* < .01) compared with WT controls (videos attached as Supporting Information File 1; Figure 4G–I). Together, the combination of altered nuclear morphology and impaired velocity parameters suggests that HIV‐1 transgene associated changes produce structurally irregular and functionally compromised sperm. These defects likely reduce fertilization efficiency in Tg animals, potentially through impaired acrosomal penetration and diminished progressive motility.

### 2.5 Impaired mitochondrial dynamics and functionality in testicular tissue of HIV‐1 Tg rats

**FIGURE 5:**
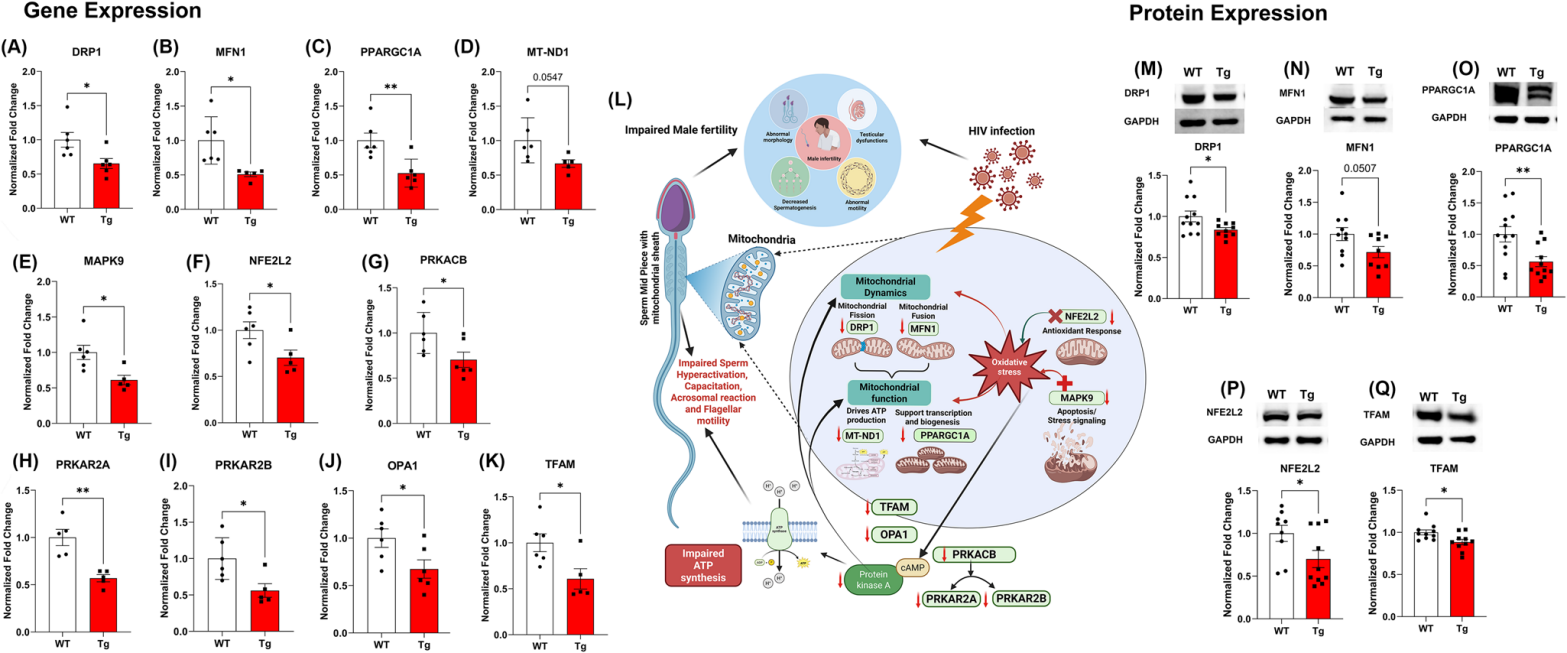
Human immunodeficiency virus (HIV)‐1 transgene disrupts mitochondrial dynamics, biogenesis and stress response signaling in transgenic (Tg) testes. Quantitative PCR (qPCR) analysis revealed significant downregulation of genes governing mitochondrial fission and fusion DRP1 and MFN1 in Tg rats compared with wild type (WT) controls (A, B). Markers of mitochondrial respiration and biogenesis were reduced, with MT‐ND1 showing a downwards trend and PPARGC1A significantly decreased (C, D). Antioxidant and stress response regulators MAPK9 and NFE2L2 were also significantly downregulated (E, F). Additional genes involved in mitochondrial signaling and genome maintenance PRKACB, PRKAR2A, PRKAR2B, OPA1 and TFAM were significantly reduced in Tg animals (G–K). (L) Proposed model summarising how impaired fission–fusion balance, reduced biogenesis and weakened antioxidant defences converge to limit ATP production, disrupt Ca^2^^+^ homeostasis and compromise sperm function. Western blot further confirmed downregulation of several of these proteins, along with a trend towards downregulation for others. DRP1, PPARGC1A, NFE2L2 and TFAM showed downregulation, while MFN1 was trending (M–Q). Data are presented as mean ± SEM; statistical significance was determined using an unpaired *t*‐test with Welch's correction. *p < .05, **p < .01. *n* = 6 samples were used for real‐time PCR (RT‐PCR) and *n* = 8–12 for Western blot.

To determine how HIV‐1 transgene impacts mitochondrial dynamics and functionality in testicular tissue, we assessed the expression of key genes involved in mitochondrial fission, fusion, biogenesis, antioxidant defence and signaling pathways. Gene expression analysis showed significant downregulation of genes governing mitochondrial structural dynamics, including DRP1 (*p* < .05) and MFN1 (*p* < .05), in Tg rats compared with WT controls (Figure 5A,B). Genes essential for mitochondrial respiration and biogenesis were also reduced. Notably, MT‐ND1 showed a downwards trend (*p* = .054), while PPARGC1A was significantly (*p* < .01) downregulated, indicating impaired mitochondrial activity and reduced capacity for biogenic renewal (Figure 5C,D). Markers of oxidative stress response and apoptotic signaling were similarly affected. MAPK9 (*p* < .05) and NFE2L2 (*p* < .05), both involved in antioxidant regulation and cellular stress mitigation, exhibited decreased expression in Tg rats, suggesting compromised oxidative defence mechanisms (Figure 5E,F). Furthermore, genes critical for mitochondrial signaling and genome maintenance including PRKACB, PRKAR2A, PRKAR2B, OPA1 and TFAM were significantly (*p* < .05) reduced in Tg animals (Figure 5G–K). Consistent with the gene expression results, protein levels of DRP1, MFN1, PPARGC1A, NFE2L2 and TFAM were significantly (*p* < .05) reduced in Tg rats relative to WT animals, whereas MFN1 was trending towards downregulation (*p* = .0507; Figure 5M–Q). The concordance between genes and protein expression data demonstrate that HIV‑1 transgene expression profoundly disrupts mitochondrial fission–fusion balance, biogenesis, antioxidant pathways and intracellular signaling in testicular tissue. Such widespread mitochondrial dysregulation likely contributes to elevated oxidative stress, diminished ATP production and impaired sperm function, ultimately promoting male infertility in Tg rats (Figure 5L).

### 2.6 Testicular inflammation in HIV‐1 transgene Tg rats

**FIGURE 6:**
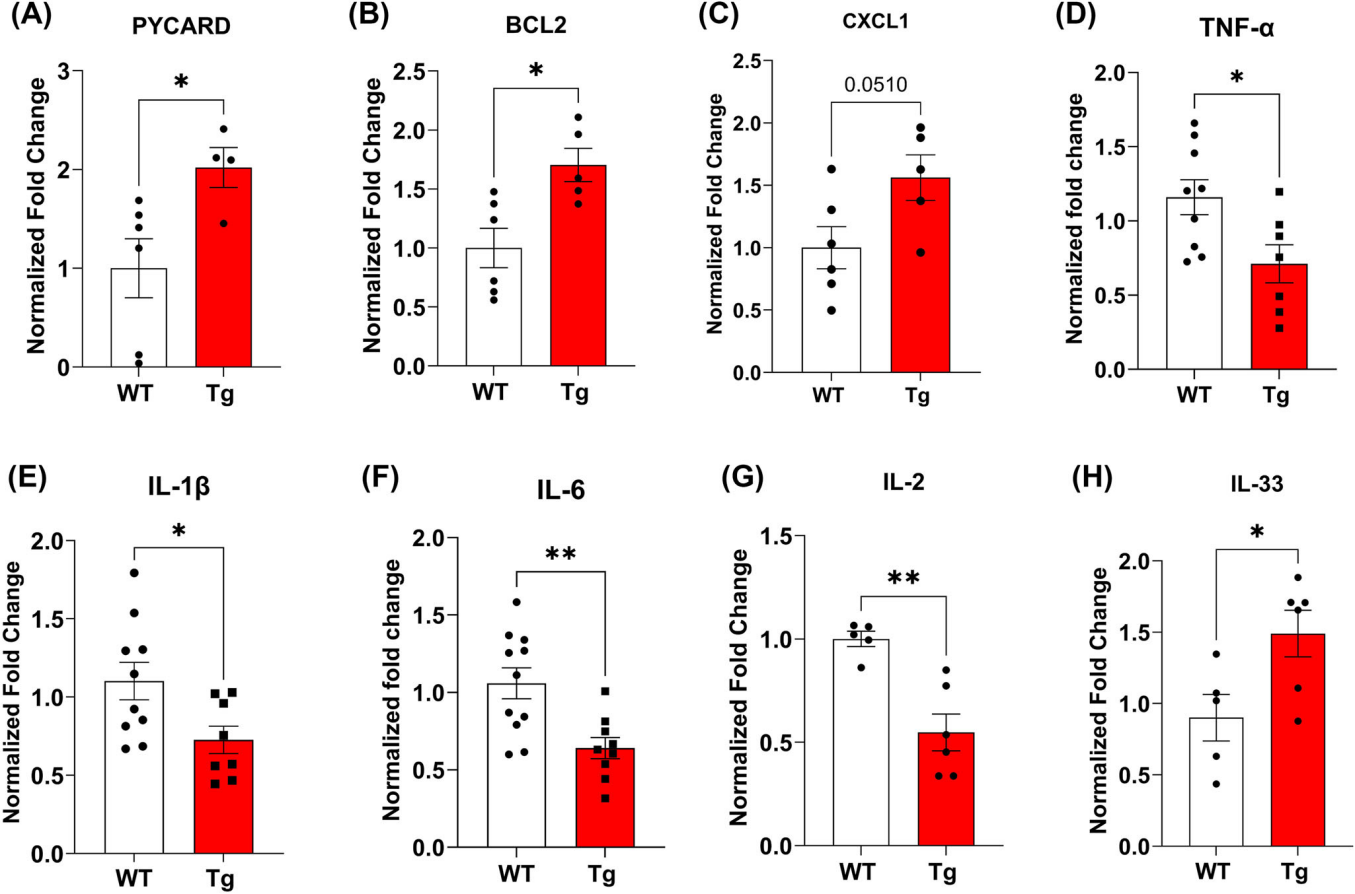
Human immunodeficiency virus (HIV)‐1 transgene affects inflammatory gene regulation in the testes. Expression of key inflammatory regulators was assessed in testicular tissue from wild type (WT) and transgenic (Tg) rats. Several genes were significantly altered in Tg animals, including upregulation of PYCARD (A) and BCL2 (B), and significant downregulation of pro‑inflammatory cytokines TNF‑α (D), IL‑1β (E) and IL‑33 (H). Prominent cytokines IL‑6 (F) and IL‑2 (G) exhibited even greater reductions in expression. In contrast, CXCL1 (C) showed a trend towards increased expression in Tg animals compared with WT. Data are presented as mean  ±  SEM, and statistical significance was determined using an unpaired *t*‐test with Welch's correction (*p* < .05; *p* < .01). *n* = 6–8 samples per group. *p < .05, **p < .01. *n* = 6–8 samples were used for the analysis.

Transcriptional profiling of testicular tissue revealed broad alterations in inflammatory signaling pathways in Tg animals compared with WT controls. PYCARD, an adaptor protein essential for inflammasome assembly, was significantly (*p* < .05) upregulated in Tg rats, indicating enhanced inflammasome activation (Figure 6A). Likewise, BCL2, a key anti‑apoptotic regulator, was significantly (*p* < .05) elevated in Tg animals, suggesting activation of survival pathways in response to cellular stress (Figure 6B).

Chemokine expression also showed notable trends. CXCL1 displayed a borderline increase (*p* = .0510) in Tg rats, indicating potential augmentation of chemokine‑mediated signaling (Figure 6C). In contrast, several classical pro‑inflammatory cytokines were significantly downregulated in Tg testes. TNF‑α, IL‑1β, IL‑6 and IL‑2 all exhibited reduced transcript levels compared with WT, with IL‑6 and IL‑2 showing highly significant (*p* < .01) decreases and TNF‑α and IL‑1β showing moderate but significant (*p* < .05) reductions (Figure 6D–H). The diminished expression of these cytokines suggests suppression of canonical inflammatory responses in Tg tissue. Together, these data indicate that HIV‑1 transgene expression is associated with a dampened classical pro‑inflammatory cytokine profile in the testes, coupled with increased expression of inflammasome‑related markers such as PYCARD and IL‑33. This combination may reflect a shift from canonical cytokine‑mediated inflammation towards inflammasome‑driven signaling, contributing to the altered immune environment observed in Tg testicular tissue.

### 2.7 RNA sequencing

#### 2.7.1 Effect of HIV‐1 transgene expression on differential gene expression in testicular tissue

**FIGURE 7:**
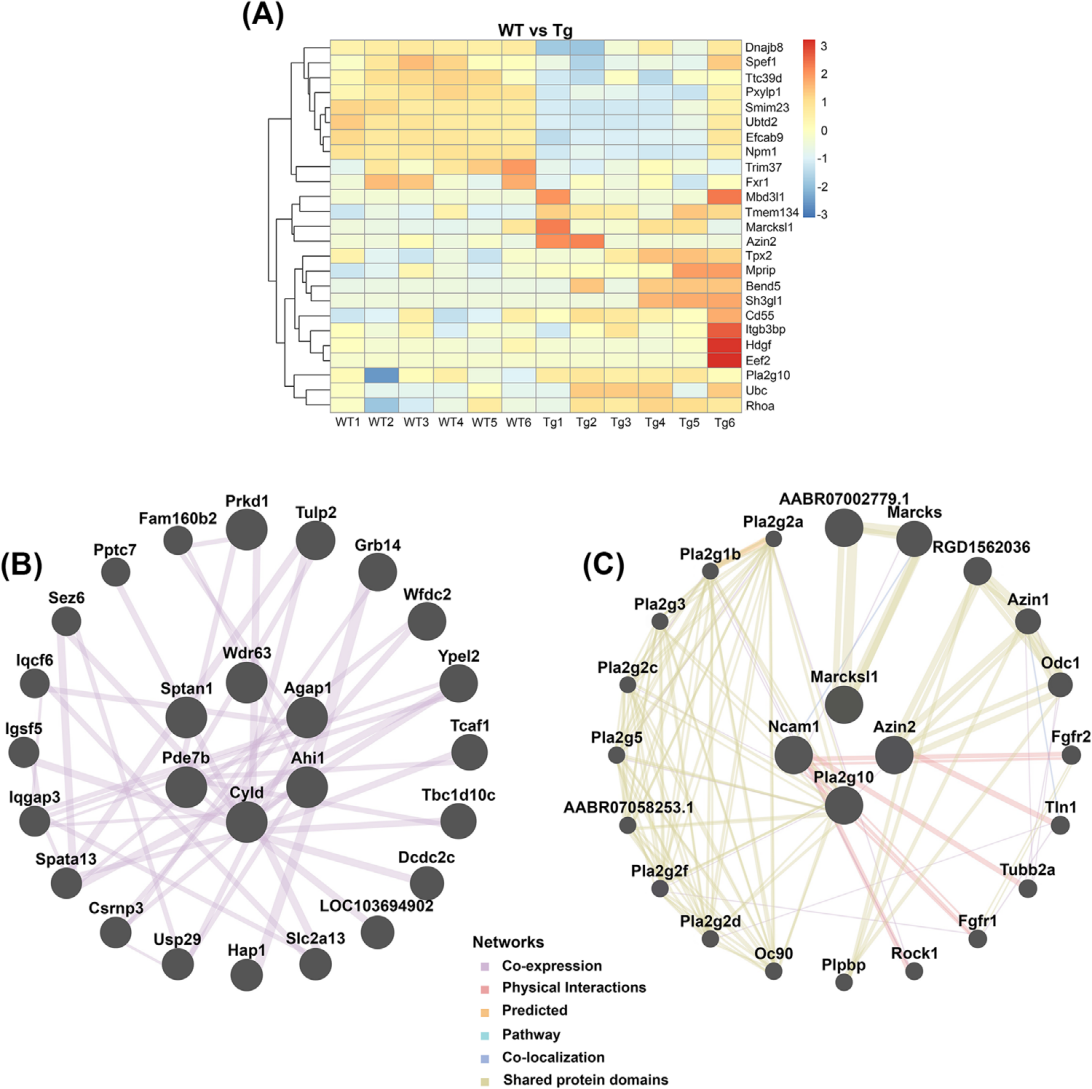
Transcriptional correlations. (A) Heatmap illustrates differential expression of inflammatory and stress‐related genes between wild type (WT) and transgenic (Tg) testes. (B, C) Gene–gene interaction networks for significantly downregulated (B) and upregulated (C) genes, highlighting coordinated repression of spermiogenic and axonemal modules (B) and activation of lipid signaling, polyamine metabolism and cytoskeletal pathways (C). Data are presented as mean ± SEM, and statistical significance was determined using an unpaired *t*‐test with Welch's correction *p < .05, **p < .01, ****p < .0001. *n* = 6 samples were used for the analysis.

RNA sequencing (RNA‐seq) analysis of testicular tissue from Tg rats compared to WT controls revealed widespread transcriptional alterations. Among the 732 differentially expressed genes (DEGs) analysed, 355 genes were significantly upregulated while 377 genes were downregulated in Tg rats, indicating a broad shift in the transcriptomic landscape. Hierarchical clustering of the top DEGs demonstrated clear segregation of Tg and WT samples, reflecting robust genotype‑dependent expression signatures (Figure 7A). Distinct expression patterns were evident, with Tg samples forming a separate cluster from WT animals, underscoring the widespread transcriptional reprogramming induced by the HIV‑1 transgene. Notably, several genes involved in chromatin regulation (TRIM37, MBD3L1), cellular stress response (DNAJB8) and signaling pathways (MARCKSL1, ITGB3BP) exhibited marked dysregulation in Tg rats. Upregulation of HDGF and EEF2, coupled with downregulation of FXR1 and TMEM134, highlights potential disruptions in testicular cellular homeostasis, protein synthesis and RNA‐binding processes. Collectively, these data demonstrate that HIV‑1 transgene expression induces substantial transcriptional remodelling in the testes. The simultaneous activation and suppression of distinct gene networks suggest widespread impacts on spermatogenesis, cell signaling and stress response pathways. These transcriptomic alterations likely contribute to the functional impairments in testicular physiology and fertility observed in Tg rats.

#### 2.7.2 Network architecture of downregulated male reproductive genes

Network analysis of significantly downregulated genes with known reproductive functions, including PDE7B, AHI1, CYLD, WDR63, SPTAN1 and AGAP1, revealed a topology driven exclusively by co‑expression relationships, indicating that HIV‑1‐associated repression affects a coordinated transcriptional program rather than disrupting a direct protein complex. Central nodes such as PDE7B, AGAP1 and SPTAN1 play essential roles in spermatogenesis signaling, vesicle trafficking and cytoskeletal integrity. Reduced PDE7B expression suggests suppression of cyclic adenosine monophosphate (cAMP)‑mediated steroidogenesis and sperm capacitation, while decreased SPTAN1, a key spectrin component involved in meiosis and flagellar assembly, implies compromised cytoskeletal remodelling during germ cell maturation. Another co‑regulated module comprising CYLD, AHI1 and WDR63 points towards defects in manchette integrity, basal‑body function and axonemal regulation. Downregulation of these genes aligns with impaired sperm tail formation, reduced flagellar motility and defective ciliary transport. Additional co‑expressed partners such as SPATA13, IQGAP3, TULP2, TCAF1 and DCDC2C further implicate disruptions in actin dynamics, calcium signaling and microtubule guidance. Collectively, the downregulated network reveals broad suppression of late spermiogenesis pathways particularly those governing axoneme assembly, structural remodeling and cAMP‑dependent activation consistent with impaired spermatid differentiation and sperm motility.

Comparing the downregulated and upregulated networks demonstrates a clear functional divergence. While downregulated genes disrupt late‐stage spermatogenic processes such as ciliary assembly, cytoskeletal maturation and activation pathways, the upregulated interactome emphasises lipid signaling, cell adhesion and polyamine‐dependent proliferation hallmarks of early and mid‐spermatogenic activity. This pattern suggests a biological shift in which early germ cell expansion is maintained or activated, whereas the structural and motility‐related machinery required for mature sperm formation is suppressed, ultimately leading to defective sperm development (Figure 7B).

#### 2.7.3 Network architecture of upregulated male reproductive genes

**FIGURE 8:**
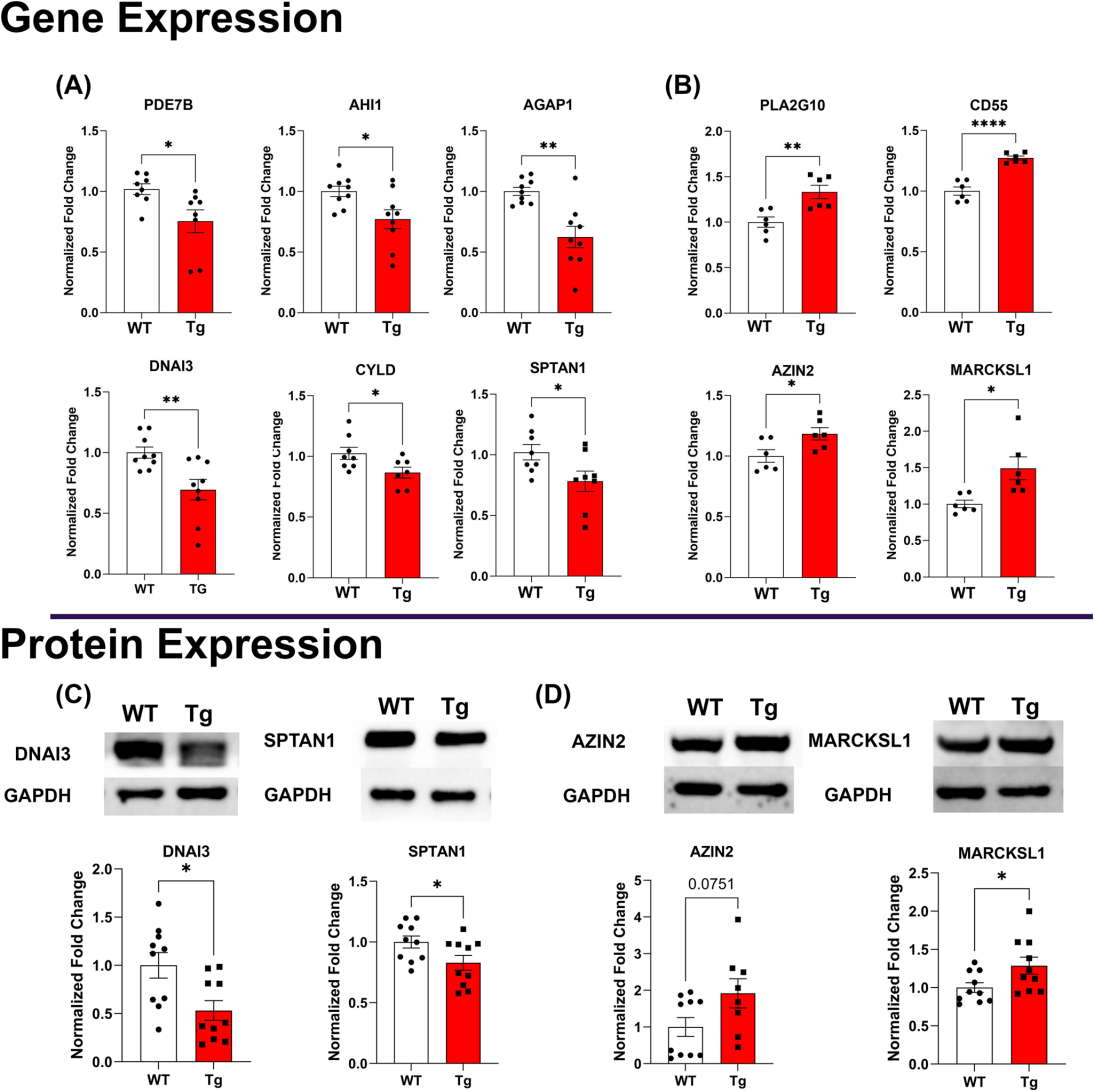
Differential expression of target genes. Real‐time PCR (RT‐PCR) validation of selected downregulated (A) and upregulated (B) genes confirms RNA‐seq trends. Western blot further supported downregulated (C) and upregulated (D) proteins. Data are presented as mean ± SEM, and statistical significance was determined using an unpaired *t*‐test with Welch's correction *p < .05, **p < .01, ****p < .0001. *n* = 6–9 for the RT‐PCR and *n* = 8–12 samples were used for the Western blot.

Integrative analysis of the top upregulated genes revealed a dense interactome consisting of lipid metabolism enzymes, polyamine regulatory factors and cytoskeletal adhesion molecules. Multiple phospholipase A2 family members PLA2G10, PLA2G2D, PLA2G2F, PLA2G3, PLA2G5, PLA2G1B and PLA2G2A formed a lipid signaling axis involved in acrosomal membrane remodeling, zona pellucida penetration and sperm–egg fusion. GeneMANIA identified strong physical interactions among these phospholipases, reflecting conserved catalytic and membrane binding domains. The second major module included polyamine‐associated genes such as AZIN2, AZIN1, ODC1 and RGD1562036, which regulate ornithine decarboxylase activity and thereby influence spermatogonial proliferation, flagellar elongation and chromatin condensation. A third subnetwork consisting of MARCKSL1, MARCKS, TLN1, TUBB2A, ROCK1 and NCAM1 highlighted enhanced cytoskeletal remodelling, Sertoli germ cell adhesion and seminiferous tubule organisation. Their interactions, supported by BioGRID and other protein interaction databases, indicate activation of integrin signaling, actomyosin contractility and structural reorganization within the testes. Together, the upregulated program suggests a shift towards lipid‐driven signaling, polyamine‐supported proliferation and cytoskeletal adaptation consistent with early spermatogenic activation (Figure 7C). Real‐time PCR (RT‐PCR) validation confirmed the RNA‐seq findings, showing significant downregulation of PDE7B (*p* < .05), AHI1 (*p* < .05), AGAP1 (*p* < .01), DNAI3 (*p* < .01), CYLD (*p* < .05) and SPTAN1 (*p* < .05), along with significant upregulation of PLA2G10 (*p* < .01), CD66 (*p* < .0001), AZIN2 (*p* < .05) and MARCKSL1 (*p* < .05) in Tg rats compared to WT controls (Figure 8A,B). To determine whether these transcriptomic alterations were reflected at the protein level, selected targets were evaluated by Western blot analysis. Consistent with the gene expression data, protein levels of DNAI3 (*p* < .05) and SPTAN1 (*p* < .05) were significantly reduced in HIV‐1 Tg animals (Figure 8C). In contrast, MARCKSL1 protein expression was significantly increased (*p* < .05), while AZIN2 exhibited a trend towards increased expression (*p* = .0751; Figure 8D). Collectively, these findings provide protein‐level validation of key transcriptomic changes identified by RNA‐seq.

#### 2.7.4 Functional enrichment analysis

**FIGURE 9:**
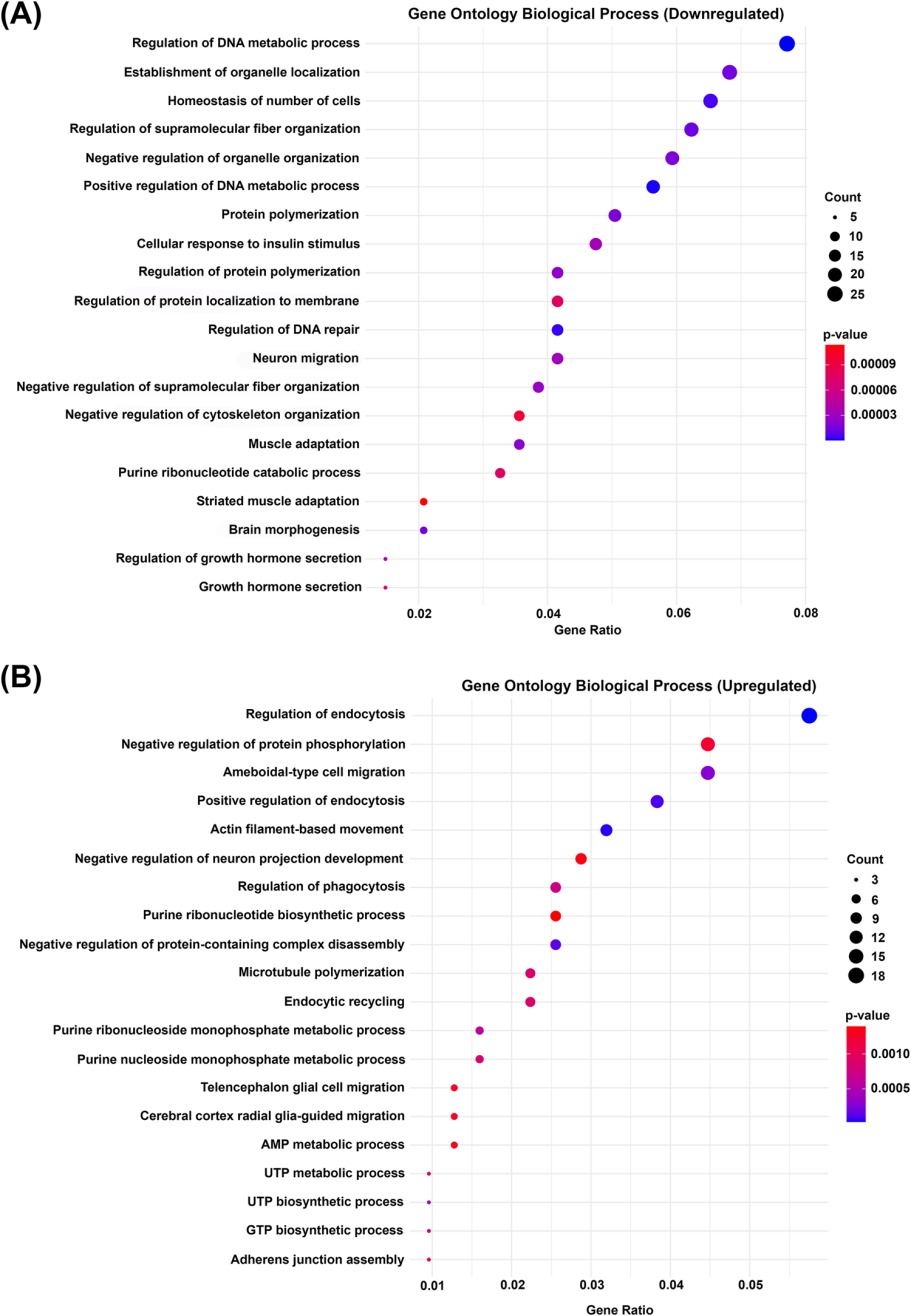
Enrichment analysis of differentially expressed genes. (A, B) Gene Ontology (GO) biological process dot plots for downregulated and upregulated gene sets. Dot size indicates gene count; colour reflects adjusted *p* values.

**FIGURE 10:**
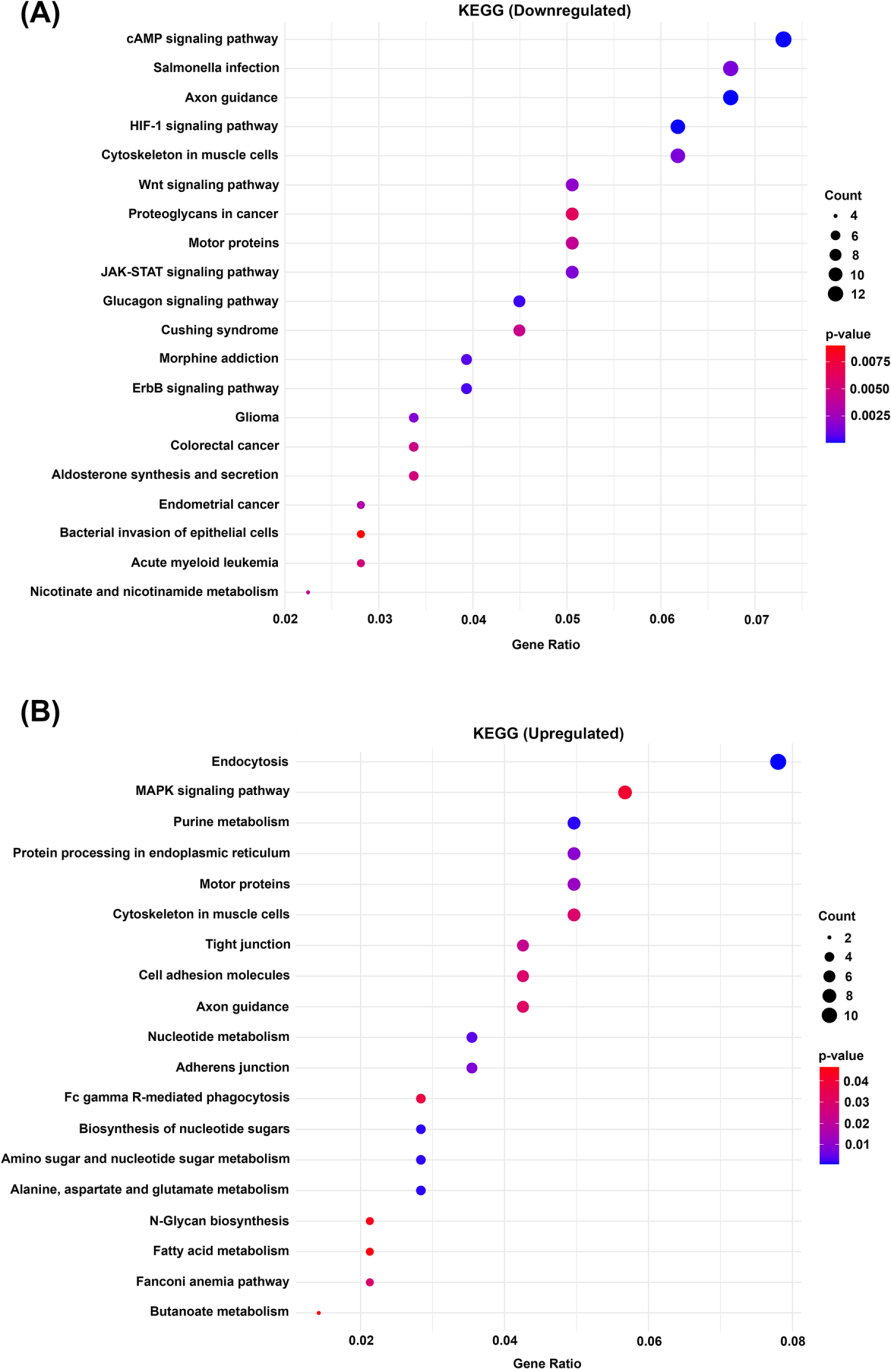
Kyoto Encyclopedia of Genes and Genomes (KEGG) pathway enrichment of differential gene sets. (A, B) Dot plots showing top enriched KEGG pathways, with dot size indicating gene count and colour representing significance (false discovery rate (FDR)‑adjusted).

Gene Ontology (GO) and Kyoto Encyclopedia of Genes and Genomes (KEGG) enrichment analyses further supported these observations. Downregulated genes were enriched in fundamental processes such as DNA metabolic regulation, chromatin organization and cell cycle control, consistent with impaired germ cell proliferation and meiotic progression. Upregulated genes were associated with protein phosphorylation, stress responses and metabolic regulation (Figure 9A,B). KEGG analysis revealed that upregulated pathways included endocytosis, mitogen‐activated protein kinase (MAPK) signaling and ER protein processing, whereas downregulated pathways were linked to JAK‐STAT signaling, apoptosis and immune regulation key components of germ cell survival and homeostasis (Figure 10A,B).

Functionally, reduced expression of SPTAN1 and DNAJB may compromise cytoskeletal stability and protein‑folding capacity, both essential for spermatid maturation. Similarly, downregulation of CYLD, a regulator of NF‑κB signaling, suggests alterations in the inflammatory microenvironment that may disrupt spermatogenesis. Decreases in central nodes such as NFE2L2 and MAPK9, which control oxidative‑stress responses and MAPK‑dependent signaling, imply weakened antioxidant defences and increased susceptibility to oxidative damage a well‑known contributor to male infertility. Altogether, these transcriptomic and network‑level alterations indicate that HIV‑1 transgene expression disrupts critical molecular pathways that regulate germ cell development, testicular homeostasis and structural maturation, ultimately contributing to reduced sperm quality, impaired motility and compromised fertility.

## 3 DISCUSSION

This study provides comprehensive evidence that HIV‐1 proviral gene expression in a Tg rat model^18^ profoundly disrupts male reproductive health through multiple mechanisms, including altered testicular morphology, hormonal dysregulation, structural and functional sperm defects, mitochondrial dysfunction, altered calcium signaling, immune dysregulation and extensive transcriptional remodeling. Importantly, this area remains underexplored in current literature, positioning these findings as novel contributions that advance understanding beyond previously published human and animal studies.

Gross morphological analysis revealed significant reductions in testicular weight and volume in Tg rats, accompanied by decreased sperm count and lower hormonal secretion including testosterone and progesterone levels. These changes are consistent with clinical reports of hypogonadism and impaired spermatogenesis in HIV‐infected men.^19^, ^20^ Notably the concomitant reductions in testosterone and progesterone levels suggest impaired testicular steroidogenesis in HIV‐1 Tg rats. When considered alongside testicular atrophy, reduced sperm counts, abnormal sperm morphology and histopathological evidence of testicular injury, these findings are consistent with primary hypogonadism resulting from intrinsic testicular dysfunction. Supporting this interpretation, FSH concentrations were comparable between groups, while LH levels were modestly elevated in Tg animals, arguing against secondary hypogonadism arising from hypothalamic or pituitary dysfunction.^21^

Histopathological analyses further revealed marked disruption of testicular architecture in HIV‐1 Tg rats, including degeneration of seminiferous tubules, reduced tubular diameter and loss of germ cell layers. The pronounced depletion of spermatocytes and spermatids indicates impaired spermatogenesis and compromised germ cell development. These structural abnormalities are consistent with previous reports of HIV‐associated testicular pathology and germ cell loss in both human and experimental studies,^22^, ^23^ further supporting the concept that chronic HIV‐1 gene expression exerts multifaceted adverse effects on male reproductive function.

Nuclear morphology analysis revealed significant alterations in Tg rats’ sperm, including reduced circularity and increased ellipticity, features that are indicative of chromatin instability and potential DNA fragmentation. Complementing these structural defects, CASA‐based motility analysis revealed significant reductions in key velocity parameters (VAP and VSL) with a significant increase in VCL, suggesting impaired sperm kinetics and compromised progressive movement. Together these morphological and functional impairments are consistent with the histopathological evidence of germ cell loss and the hormonal deficits observed in HIV‐1 Tg animals. Collectively, the findings suggest a marked impairment in reproductive capacity, with defects in sperm quality, testicular function and spermatogenesis likely contributing to reduced fertilization potential and compromised male fertility.

A key novel finding of this study is the marked downregulation of CatSper subunits (CatSper1–4) in HIV‐1 Tg rats, indicating a disruption of the CatSper channel complex. CatSper channels are sperm‐specific Ca^2^^+^ channels localized to the principal piece of the flagellum, where their activation by intracellular alkalinisation mediates the Ca^2^^+^ influx required for sperm hyperactivation, chemotaxis and fertilization.^24^ Because CatSper1–4 constitute the pore‐forming α subunits essential for channel function, the coordinated reduction in expression of all four subunits observed in Tg rats suggests impaired CatSper‐dependent Ca^2^^+^ signaling. This observation is consistent with previous studies demonstrating that deficiency or dysfunction of CatSper α subunits, particularly CatSper1 and CatSper2, results in impaired sperm hyperactivation and male infertility.^25^

Importantly, the CatSper deficit identified in our results aligns closely with the marked reductions in progesterone and testosterone levels observed in Tg animals. Progesterone is a well‐established physiological activator of CatSper channels and drives rapid Ca^2^^+^ influx through this complex,^26^ while testosterone supports Sertoli cell function, spermatogenesis and mitochondrial bioenergetics.^27^ The observed the hormonal disturbances in Tg rats may exacerbate CatSper dysfunction, creating a multifaceted impairment of sperm function. Reduced testosterone may compromise mitochondrial integrity and ATP production, while diminished progesterone signaling may impair CatSper‐mediated calcium influx. Together, these alterations are likely to contribute to the mitochondrial dysfunction and motility deficits observed in HIV‐1 Tg animals.

Importantly, the observed reduction in CatSper transcript levels was corroborated by decreased CatSper protein expression, providing independent validation of CatSper dysregulation in the testes of HIV‐1 Tg rats. Collectively, these findings suggest that impaired CatSper signaling may represent a key molecular mechanism linking the hormonal, mitochondrial and transcriptional abnormalities associated with HIV‐1 transgene expression, ultimately contributing to compromised sperm function and male reproductive health.

Mitochondria are central to sperm physiology, providing ATP for motility and regulating calcium homeostasis, which is essential for hyperactivation and acrosome reaction.^28^ In this study, Tg rats showed a profound disruption of mitochondrial dynamics and function in the testicular tissue. We observed significant downregulation of DRP1 and MFN1, key regulators of mitochondrial fission and fusion, respectively. This imbalance likely leads to fragmented or excessively fused mitochondria, impairing energy distribution along the sperm tail and reducing motility. Furthermore, decreased expression of PPARGC1A and TFAM, master regulators of mitochondrial biogenesis and transcription, suggests a reduction in mitochondrial number and compromised mitochondrial DNA maintenance.^29^ These deficits would severely restrict oxidative phosphorylation capacity, leading to reduced ATP production and impaired support for sperm motility, capacitation and overall reproductive competence.

The downregulation of MT‐ND1, a core component of Complex I in the electron transport chain, indicates impaired electron flow and ATP synthesis. Such dysfunction promotes electron leakage and reactive oxygen species (ROS) generation, exacerbating oxidative stress. Reduced expression of antioxidant regulators NFE2L2 and MAPK9 at both transcript and protein levels further supports impaired cellular stress responses and is consistent with mitochondrial dysregulation.^30^, ^31^, ^32^ Together, these changes create a pro‐oxidative environment within the testes, contributing to lipid peroxidation of sperm membranes, DNA fragmentation and protein damage all which are known drivers of abnormal sperm morphology and reduced fertilization potential.

Mitochondrial impairment also affects intracellular calcium buffering, as they are essential regulators of Ca^2^^+^ uptake and release. Disruption of this regulatory capacity may be associated with altered CatSper channel expression, and impaired sperm function, although a direct relationship remains to be experimentally established.^33^ The convergence of mitochondrial impairment, oxidative stress and disrupted calcium signaling provides a mechanistic basis for the reduced progressive motility, abnormal nuclear morphology and decreased CatSper expression seen in Tg animals. Collectively, these findings position mitochondria as a central hub linking energy depletion, ROS accumulation and Ca^2^^+^ dysregulation three interdependent processes that culminate in impaired sperm function and male infertility.^34^

Inflammatory profiling revealed a complex and dysregulated immune signature in HIV‐1 Tg testes, characterised by increased expression of inflammasome‐associated genes (PYCARD and BCL2) alongside reduced expression of key pro‐inflammatory cytokines (TNF‐α, IL‐1β, IL‐6 and IL‐2). Rather than reflecting a classical inflammatory response, this pattern suggests chronic immune remodelling within the testicular microenvironment, potentially driven by persistent HIV‐1 transgene expression, seminiferous tubule degeneration, loss of germ cells and compensatory immunoregulatory mechanisms. The reduced cytokine expression may indicate impaired immune signaling within damaged testicular tissue, whereas elevated inflammasome‐related markers are consistent with ongoing cellular stress and innate immune activation.

These molecular alterations closely parallel the histopathological findings, including seminiferous tubule degeneration, depletion of germ cell layers and widespread disruption of testicular architecture, suggesting compromised testicular homeostasis and spermatogenesis. Collectively, the data support the presence of a dysregulated testicular immune microenvironment that may contribute to reproductive dysfunction in HIV‐1 Tg rats. However, additional studies incorporating cell‐specific profiling and protein‐level analyses of inflammatory mediators will be required to distinguish between chronic immune remodelling, immune suppression and changes in tissue cellular composition.

RNA‐seq analysis revealed extensive transcriptional reprogramming in Tg testes, with 355 genes upregulated and 377 downregulated, reflecting a broad disturbance of testicular homeostasis driven by HIV‐1 proviral gene expression. Downregulated genes were strongly enriched for pathways governing spermatid differentiation, axonemal assembly, cytoskeletal remodeling and cAMP‐mediated activation core processes required for sperm motility, capacitation and hyperactivation. These transcriptional deficits directly align with the observed motility reductions in VSL and VAP and increase in VCL, suggesting that suppression of late spermatogenic gene networks translates into functional impairment of the mature sperm cell. Moreover, genes such as PDE7B, CYLD and AHI1, which serve as key molecular hubs regulating flagellar structure, manchette organisation and sperm activation, were significantly downregulated. Their suppression provides mechanistic insight into the distorted sperm nuclear morphology, defective tail assembly and reduced fertilizing ability observed in Tg animals.

In contrast, upregulated genes clustered around lipid metabolic pathways, polyamine biosynthesis and cytoskeletal remodeling hallmarks of metabolic reprogramming commonly observed under cellular stress. The enrichment of phospholipase A2 family members suggests enhanced membrane turnover or compensatory remodeling of germ cell membranes in response to cellular damage. Upregulation of polyamine‑regulatory genes such as AZIN2 and ODC1 implies activation of proliferative programs and altered chromatin remodeling, potentially reflecting attempts by the testes to maintain spermatogonial or early spermatocyte viability. However, even as metabolic pathways are upregulated, the failure of structural and motility‑related genes to activate suggests a decoupling between growth/proliferation signals and terminal differentiation hallmark of dysfunctional spermatogenesis.

Collectively, these findings illustrate a multifactorial and interdependent mechanism by which HIV‑1 proviral gene expression impairs male fertility. Hormonal disruption specifically reduced testosterone and progesterone compromises Sertoli cell support, spermatogenesis, mitochondrial biogenesis and CatSper channel activation. Histopathological degeneration, including seminiferous tubule thinning, loss of germ cell layers and reduced Leydig cell density, indicates severe architectural destabilization of the reproductive niche. Mitochondrial dysfunction further compounds these defects by limiting ATP production, elevating ROS and impairing Ca^2^^+^ buffering, thereby damaging DNA, proteins and membranes while disrupting hyperactivation signaling. Downregulation of the CatSper complex the central Ca^2^^+^ channel for sperm motility directly inhibits Ca^2^^+^ influx necessary for hyperactivation, chemotaxis and acrosome reaction, creating a profound block at the level of sperm function.

Transcriptional remodelling appears to be a central mechanism underlying the reproductive dysfunction observed in HIV‐1 Tg rats. Suppression of genes involved in spermatid differentiation and sperm maturation, together with activation of metabolic and cellular stress pathways, suggests a maladaptive response that fails to restore normal testicular function. These transcriptional alterations are accompanied by evidence of immune dysregulation within the testes, characterised by reduced expression of classical pro‐inflammatory cytokines and increased expression of inflammasome‐associated markers. This atypical immune profile is consistent with chronic immune remodeling and persistent cellular stress rather than a conventional inflammatory response. Such alterations may disrupt testicular homeostasis, impair germ cell survival and compromise the microenvironment required for normal spermatogenesis.

Within this framework, the CatSper dysregulation emerges as a potentially important component linking hormonal, mitochondrial and transcriptional alterations observed in HIV‐1 Tg rats. Given the essential role of CatSper channels in sperm physiology, reduced expression may contribute to the motility defects observed in Tg animals. Nevertheless, further studies evaluating CatSper channel function, calcium signaling and sperm hyperactivation are required to define its precise mechanistic contribution.

Overall, the combined transcriptomic, morphological, biochemical and functional data from this study present a compelling case that HIV‐1 proviral gene expression disrupts male fertility through a complex, multilayered cascade. By integrating hormonal imbalance, mitochondrial dysfunction, calcium‐channel impairment, immune dysregulation and transcriptional rewiring, this work provides one of the most comprehensive mechanistic frameworks to date for understanding HIV‐associated reproductive impairment. These findings not only advance the field significantly but also highlight critical molecular targets, especially CatSper, that may inform the development of therapeutic strategies aimed at preserving fertility in individuals living with HIV.

The HIV‐1 Tg rat is a well‐established and extensively validated model that has been widely used to investigate the chronic consequences of persistent HIV‐1 gene expression across multiple organ systems. Because the model exhibits sustained expression of HIV‐1 proteins in the setting of minimal to no productive viral replication, it closely reflects an important aspect of contemporary HIV disease, namely, the persistence of viral gene products and chronic immune dysregulation despite effective virologic suppression. As such, it has provided valuable insights into HIV‐associated comorbidities, including neurological, metabolic, cardiovascular, renal and reproductive complications.

At the same time, several considerations should be acknowledged when interpreting the present findings. The model does not incorporate ART exposure, progressive immune deficiency or the broader clinical heterogeneity observed among people living with HIV. Moreover, ART itself has been reported to influence sperm quality, mitochondrial function and reproductive outcomes. Therefore, while the HIV‐1 Tg rat offers a highly relevant and widely accepted platform for investigating mechanisms of chronic HIV‐associated reproductive dysfunction, caution is warranted when extrapolating these findings directly to clinical populations. Future studies incorporating ART exposure and additional clinically relevant variables will be important for further defining the translational significance of the molecular and reproductive alterations identified in this study.

## 4 CONCLUSION

In summary, our findings support a multifactorial model in which chronic HIV‐1 gene expression disrupts male reproductive function through coordinated alterations in testicular structure, spermatogenesis, sperm quality and molecular signaling pathways. HIV‐1 Tg rats exhibited testicular degeneration, impaired spermatogenesis, abnormal sperm morphology and reduced sperm motility, accompanied by marked transcriptional and protein‐level changes affecting CatSper signaling, mitochondrial homeostasis and immune regulation. The downregulation of CatSper1–4, together with evidence of mitochondrial dysfunction and altered expression of genes involved in spermatid differentiation and axonemal assembly, suggests disruption of pathways critical for sperm maturation and function. Collectively, these findings provide new insight into the molecular alterations associated with HIV‐related reproductive dysfunction and identify CatSper signaling, mitochondrial function and immune dysregulation as important pathways for further investigation. Future studies incorporating functional, hormonal, oxidative stress and interventional approaches will be necessary to determine the causal significance of these alterations and their relevance to male reproductive health in the context of HIV infection.

## 5 MATERIALS AND METHODS

### 5.1 Animals

Male Fischer 344 WT and F344 Hsd: HIV‐1 Tg rats aged 10–12 weeks old were used in this study.^18^ The Tg rats express an HIV‐1 proviral transgene lacking the gag and pol regions. All animals were bred under appropriate licensure and housed under a 12‐h light/dark cycle with ad libitum access to food and water. A total of 8–12 animals per group (WT and Tg) were included. All procedures were approved by the Institutional Animal Care and Use Committee (IACUC) at the University of Nebraska Medical Center (protocol 22‐081‐03‐FC, approved 11/20/2025) and were conducted in accordance with the NIH Guide for the Care and Use of Laboratory Animals.

### 5.2 Rat sperm collection and purification

Sperm collection from the rat epididymis was performed with minor modifications to previously described methods.^35^ Both cauda epididymides were carefully dissected, minced with fine scissors and immersed in 1 mL of M2 medium pre‑warmed to 37°C (M7167, Sigma‑Aldrich). The tissue was incubated at 37°C for 10 min to allow motile sperm to swim out. The resulting sperm suspension was collected and centrifuged at 2700 × *g* for 4 min at room temperature. The sperm pellet was washed twice with double‑distilled water (ddH_2_O), centrifuging again at 2700 × *g* for 4 min between washes. The final purified sperm pellet was then used for downstream analyses.

### 5.3 CASA and sperm cell counts

Motile sperm collected from the cauda epididymides and incubated in M2 medium at 37°C for 10 min were placed onto microscope slides and recorded using an AmScope MU1003 camera. Video recordings were analysed with the ImageJ CASA plugin developed by Wilson‑Leedy and Ingermann.^36^, ^37^ Total sperm cells were determined using a Neubauer hemocytometer following WHO guidelines.^38^

### 5.4 Nuclear morphology

For nuclear morphology analysis, sperm cells were smeared on a glass slide, fixed with 4% paraformaldehyde (PFA) for 15 min, and air‐dried. Following fixation, slides were incubated with DAPI‐stain (.3 mM; Cat# D9542) for 30 min and then washed with phosphate‐buffered saline (PBS). After air‑drying, slides were mounted using ProLong™ Antifade (Invitrogen P36930). Images were captured at 60× magnification using an Invitrogen EVOS M5000 microscope. Nuclear morphology was quantified using the analysis software developed by Skinner et al. to evaluate sperm head shape characteristics.^39^

### 5.5 Quantification of progesterone and testosterone by ELISA

Plasma progesterone and testosterone concentrations were quantified using competitive ELISA assays: RayBiotech Progesterone ELISA Kit (Cat# EIA P4) and Enzo Life Sciences Testosterone ELISA Kit (Cat# ADI 900 065). All procedures were performed according to the manufacturers’ instructions with minor modifications described below.

Blood was collected into EDTA tubes and centrifuged at 1500 × *g* for 10 min at 4°C; plasma was aliquoted and stored at −80°C until use. Before assay, samples were thawed on ice and diluted 1:5–1:20 depending on kit requirements. Standards and diluted plasma (50–100 µL) were added to antibody‑coated wells along with enzyme conjugates and incubated according to manufacturer protocols. After washing (3–5×), TMB substrate was added, incubated in the dark and the reaction stopped with 1 M sulphuric acid. Absorbance was recorded at 450 nm. Because both assays use a competitive format, absorbance is inversely proportional to hormone concentration. Standard curves were generated using a four‑parameter logistic (4‑PL) model, and hormone concentrations were interpolated accordingly. All samples were run in duplicate.

### 5.6 RNA isolation and real‐time quantitative PCR array

Total RNA was isolated from testicular tissue using the Direct‐zol RNA MiniPrep Plus kit (Zymo Research Corp.), following the manufacturer's instructions. Purified RNA was stored at −80°C until used in gene expression analyses. cDNA was synthesised from total RNA using the SuperScript™ IV Reverse Transcriptase kit (Invitrogen). qPCR was performed on a QuantStudio™ 7 Flex Real‑Time PCR System (Applied Biosystems). CatSper1–4 gene expression was assessed using TaqMan assays obtained from Applied Biosystems (Cat# 4351372, 4331182, 4331182, 4351372). Gene expression levels were normalized to a 1:100 dilution of 18S rRNA as the endogenous control, and relative quantification was calculated using the ΔΔCt method.^40^

### 5.7 Histopathology

Testes from both experimental groups were isolated and fixed in Modified Davidson's Fixative (Cat# 64133‑50) for 72 h before undergoing routine tissue processing. Fixed tissues were dehydrated through a graded ethanol series, cleared in xylene, infiltrated with paraffin and embedded. Serial sections of 5 µm thickness were cut using a Leica rotary microtome. Sections were stained with haematoxylin and eosin (H&E) and imaged using a Hamamatsu NanoZoomer S360 slide scanner. Digital images were examined and analysed using Aperio ImageScope software (Version 12.4.6.5003).

### 5.8 Western blotting

Testicular tissues were homogenized in Tissue Protein Extraction Reagent (T‐PER; Thermo Fisher Scientific) supplemented with 1% protease and phosphatase inhibitor cocktail (Thermo Fisher Scientific). Homogenates were mechanically disrupted, sonicated and centrifuged at 300 × *g* for 5 min at 4°C. Total protein concentrations were determined using the Pierce bicinchoninic acid (BCA) protein assay kit (Thermo Fisher Scientific).

Equal amounts of protein were resolved under reducing conditions on 4%–12% or 10% Bis–Tris polyacrylamide gels (Invitrogen) and transferred onto nitrocellulose membranes using the iBlot™ 2 transfer system (Invitrogen). Membranes were stained with Ponceau S (Sigma‐Aldrich) to verify protein transfer and equal loading, followed by blocking in 5% nonfat dry milk at room temperature for 1–2 h. Membranes were then incubated overnight at 4°C with primary antibodies and subsequently with the appropriate secondary antibodies following standard washing procedures.

Protein bands were visualized using Radiance ECL or Radiance Plus chemiluminescent substrates (Azure Biosystems) and imaged with an Azure c300 imaging system. Detailed information regarding antibodies, protein loading amounts and dilution conditions is provided in Supporting Information File 3, Table S1. Band intensities were quantified using ImageJ software (version 1.54j; National Institutes of Health).^41^

### 5.9 RNA‐seq data processing and functional enrichment

RNA‐seq data from testicular tissues of WT and HIV‐1 Tg rats (*n* = 6 per group) were processed in R to identify DEGs. Differential expression analysis was performed using established RNA‐seq pipelines, and genes were classified as upregulated or downregulated based on fold‐change criteria (≥2 or ≤−2) and statistical significance (*p* < .05), with only significant DEGs retained for downstream interpretation.^42^, ^43^ Ensemble gene identifiers were converted to Entrez Gene IDs using the org.Rn.eg.db annotation package for Rattus norvegicus, and duplicate mappings were removed to ensure unique gene assignments.^44^ Functional enrichment analyses were conducted separately for upregulated and downregulated gene sets using GO (biological process, cellular component and molecular function), KEGG, Reactome and WikiPathways. Multiple‐testing correction was applied using the Benjamini–Hochberg method, and enriched pathways were ranked by adjusted *p* value, with the top terms selected for interpretation and visualization.^45^, ^46^, ^47^, ^48^

### 5.10 Gene–gene interaction network construction

Gene–gene interaction networks were constructed separately for the downregulated and upregulated DEGs with annotated reproductive function using the GeneMANIA prediction server (https://genemania.org; accessed [7/2026]).^49^, ^50^ Analyses were performed using the *Rattus norvegicus* database with default search settings.

Network construction incorporated all available interaction categories, including co‐expression, physical interactions, genetic interactions, co‐localization, pathway associations, predicted interactions and shared protein domains. GO‐derived functional interaction networks were excluded, consistent with GeneMANIA default settings, to minimise potential circularity arising from GO‐based functional annotation. Co‐expression evidence was restricted to the twenty most informative datasets included in the default configuration, and co‐expression links were retained only where independently supported by at least one additional network. A maximum of twenty functionally related genes were permitted to be added to each query set; attribute nodes were not included.

Network weighting was performed using GeneMANIA's default automatic weighting algorithm. For query gene sets containing five or more genes, GeneMANIA applies the assigned based on query genes method, in which network weights are calculated using linear regression to maximise connectivity among the input genes. Networks that do not contribute to the connectivity of the query gene set are assigned a weight of zero and excluded from the final composite network. Interaction data were derived from curated resources integrated within GeneMANIA, including BioGRID, IRefIndex, Pathway Commons, I2D and GEO‐based co‐expression datasets.

Unlike STRING, GeneMANIA reports network weights rather than confidence scores. No additional weight thresholds or post hoc edge filtering were applied; therefore, all interactions included in the composite network were retained for visualization. In the resulting networks, node size reflects the GeneMANIA gene score, edge thickness corresponds to the normalized interaction weight, and edge color indicates the underlying interaction category.

### 5.11 Gene expression visualization and computational environment

To assess global expression patterns, the top 25 DEGs were selected for heatmap visualization.^51^ Expression values were log2‐transformed (FPKM + 1) to normalize distributions, and row‐wise scaling was applied to emphasise relative differences across samples.^52^, ^53^, ^54^ Genes were hierarchically clustered, while sample clustering was disabled to preserve WT and Tg group structure (WT1–WT6 and Tg1–Tg6). All analyses were performed in R (version 4.4.2) using established packages for data processing, functional enrichment and visualization.

### 5.12 Statistical analysis

Statistical analyses were performed using GraphPad Prism (Version 10.6; GraphPad Software). Data are presented as mean ± SEM. Group comparisons were evaluated using an unpaired *t*‐test with Welch's correction to account for unequal variances. A *p* value ≤.05 was considered statistically significant.

## AUTHOR CONTRIBUTIONS

Tousif Ahmed Hediyal and Gurudutt Pendyala conceptualised and designed research; Tousif Ahmed Hediyal, Elizabeth M. Stone, Omar Shukri, Victoria L. Schaal, Amin Foroughi‐Nezhad, Murali Devanaboyina, Josef S. Crenshaw, Amir Elrefaie and Sree Kolli performed research; Dinesh Pradhan and Sowmya V. Yelamanchili contributed new reagents/analytic tools; Tousif Ahmed Hediyal, Elizabeth M. Stone, Omar Shukri, Victoria L. Schaal, Amin Foroughi‐Nezhad, Murali Devanaboyina, Sree Kolli and Camryn N. Davis analysed data; Sowmya V. Yelamanchili and Gurudutt Pendyala edited the paper; and Tousif Ahmed Hediyal, Elizabeth M. Stone, Omar Shukri and Amin Foroughi‐Nezhad wrote the paper.

## ETHICS STATEMENT

All animal procedures were approved by the Institutional Animal Care and Use Committee (IACUC) at the University of Nebraska Medical Center (protocol 22‐081‐03‐FC, approved 11/20/2025) and were conducted in accordance with the NIH Guide for the Care and Use of Laboratory Animals

## CONFLICT OF INTEREST STATEMENT

The authors declare no conflicts of interest.

## Supporting information



Overview of sperm motility differences between WT and Tg males. This movie presents real‑time recordings of sperm motility captured under identical imaging conditions. Sperm from WT males exhibit progressive, coordinated forward movement, whereas sperm from Tg males show diminished progressive motility accompanied by altered flagellar beating patterns. All videos were recorded immediately after capacitation and are displayed at real‑time speed.

Supporting Information

This file represents the list of abbreviations which have been used in the manuscript.

Summary of antibodies used for Western blot analysis, including protein loading information, manufacturer's details, catalogue numbers and dilutions ratios.

Original uncropped Western blot images used for all the protein analyses in the manuscript. Boxed region denotes the bands included in the corresponding figure and used for densitometric quantification.

Detailed description of RNA library preparation, sequencing, quality control, read mapping, normalization, differential expression analysis and DEG selection criteria.

## Data Availability

Raw RNA‐sequencing data have been deposited in the NCBI Sequence Read Archive (SRA) under BioProject accession PRJNA1428281. Corresponding BioSample accessions are SAMN55952246–SAMN55952257. Processed gene expression matrices and associated metadata will be deposited in the Gene Expression Omnibus (GEO) and released upon publication. All analysis workflows and scripts are available from the corresponding author upon reasonable request.
