# Supplementary material for: Testicular toll with viral intrusion: Molecular insights into the impact of chronic HIV infection on male fertility
Source: Clin Transl Med. 2026 Sep 11;16(9):e70815. doi: 10.1002/ctm2.70815 (PMC13563373; doi:10.1002/ctm2.70815)
Supplement: Supplementary file 1 [file CTM2-16-e70815-s005.docx]

**RNA Extraction, Library Preparation, and Sequencing**

Total RNA was isolated from testicular tissue of wild-type and HIV-1 transgenic rats (n = 6 per group) using the Direct-zol RNA MiniPrep Plus kit (Zymo Research, Irvine, CA, USA) according to the manufacturer's instructions. RNA integrity was assessed on an Agilent 2100 Bioanalyzer (Agilent Technologies, Santa Clara, CA, USA). Poly(A)-selected libraries were prepared by LC Sciences (Houston, TX, USA) following the Illumina TruSeq stranded mRNA protocol. Poly(A)-containing transcripts were enriched using oligo-(dT) magnetic beads through two rounds of purification, fragmented in divalent cation buffer at elevated temperature, and reverse-transcribed into double-stranded cDNA. Final libraries were quality-checked and quantified on an Agilent 2100 Bioanalyzer High Sensitivity DNA chip, and paired-end sequencing was performed on an Illumina NovaSeq 6000 platform.

Sequencing generated 36.7–53.7 million raw reads per sample (5.5–8.1 Gb). Following removal of adapter sequences, low-quality bases, and undetermined bases with Cutadapt [new ref] and in-house Perl scripts, 35.5–52.0 million high-quality reads per sample were retained (5.3–7.8 Gb; 96.3–97.0% of raw reads). Post-filtering read quality was verified with FastQC (v0.10.1; <https://www.bioinformatics.babraham.ac.uk/projects/fastqc/>); Q20 exceeded 99.99% and Q30 ranged from 99.06% to 99.14% across all twelve libraries, with GC content of 50.5%.

Clean reads were aligned to the *Rattus norvegicus* reference genome (Ensembl release 112, assembly mRatBN7.2) using HISAT2 (v2.0). Overall alignment rates ranged from 96.9% to 97.2%, with 93.9–94.2% of reads mapping uniquely and 2.9–3.3% mapping to multiple locations; 89.1–90.3% of read pairs aligned concordantly. Reads distributed approximately evenly between the sense and antisense strands (≈47% each), consistent with the stranded library chemistry. Aligned reads were assembled per sample with StringTie (v1.3.4) [new ref], individual assemblies were merged into a unified transcriptome using gffcompare, and transcript- and gene-level abundances were estimated with StringTie and the ballgown package as FPKM (fragments per kilobase of transcript per million mapped reads).

**RNA-seq Data Processing and Functional Enrichment**

Differential expression analysis between wild-type and HIV-1 transgenic testes was performed on gene-level counts using DESeq2 [42], which applies a median-of-ratios size-factor normalization to correct for differences in library depth and composition and models counts under a negative binomial distribution with empirical Bayes shrinkage of dispersion estimates; edgeR [43] was used for supporting pairwise comparisons. Multiple testing was addressed using the Benjamini-Hochberg false discovery rate procedure [50].

*Genes were defined as differentially expressed if they met a nominal p < 0.05 together with an absolute fold change ≥ 2 (|log₂ fold change| ≥ 1). This yielded 732 DEGs, of which 355 were upregulated and 377 were downregulated in transgenic relative to wild-type testes. Because this threshold is based on unadjusted p-values, the DEG set is intended as a discovery-level catalogue for pathway-level interpretation and network analysis rather than as a set of individually FDR-controlled findings; accordingly, ten transcripts were independently validated by RT-qPCR and four at the protein level by Western blot.*

Ensembl gene identifiers were converted to Entrez Gene IDs using the org.Rn.eg.db annotation package for *Rattus norvegicus* [44], and duplicate mappings were removed to ensure unique gene assignments. Functional enrichment was conducted separately for the upregulated and downregulated gene sets against Gene Ontology (biological process, cellular component, molecular function), KEGG [45], Reactome [48], and WikiPathways [46] using clusterProfiler [47] and ReactomePA [48]. Enrichment p-values were corrected by the Benjamini-Hochberg method, and pathways were ranked by adjusted p-value, with top terms selected for interpretation and visualization.
